# Like humans, language models demonstrate face-to-character biases

**DOI:** 10.1093/pnasnexus/pgag247

**Published:** 2026-08-18

**Authors:** Steven A Lehr, Yash Lothe, Mahzarin R Banaji

**Affiliations:** Cangrade, Inc., 9 Munroe Ave., Watertown, MA 02472, USA; Language Technologies Institute, School of Computer Science, Carnegie Mellon University, Pittsburgh, PA 15213, USA; Department of Psychology, Harvard University, Cambridge, MA 02138, USA

**Keywords:** large language models (LLMs), machine psychology, algorithmic bias, face biases, multimodal models

## Abstract

Humans routinely make unjustified character inferences, such as labeling people as *trustworthy* or *untrustworthy*, based on facial features. We conducted 13 experiments, using four models and totaling nearly 8,000 trials, to ask: Would large language models (LLMs), trained foundationally on language and not images, mirror human biases by inferring character traits from 2D facial images, or would they be free from this human error? GPT-4o reliably exhibited face-biased judgments of *competence* and *trustworthiness* (experiments 1 and 2), generalized these judgments to semantically related traits (experiments 3 and 4), and even to primate faces that were absent from its training data (experiment 5). Strikingly, the LLM's face-to-character inferences escalated to ascriptions of extreme negative behavior such as murder and human trafficking (experiment 6) as well as to positive high-impact decisions like selection for employment or venture capital funding (experiment 7). To ensure these results were not an idiosyncratic feature of a particular LLM (GPT-4o), in experiments 8–13, we showed the same patterns, usually with an even greater degree of bias, in GPT-5, Gemini 3 Flash Preview, and Claude Sonnet 4.5. These robust biases stand in contrast to LLMs’ reluctance to explicitly endorse race/gender stereotypes, and suggest that alignment efforts to date have been domain-specific, with current models lacking generalized egalitarian decision-making.

Significance Statement As language models become increasingly entrenched in human decisions, ensuring the neutrality and accuracy of their judgments is crucial. We uncovered a surprising rupture in this neutrality when large language models (LLMs) assessed character from 2D facial images. Instead of refusing the requests or answering neutrally, all four tested LLMs made incorrect inferences about individuals’ trustworthiness and/or competence based on facial features. Moreover, they readily incorporated these errors into consequential good and bad decisions, ranging from worthiness of venture capital funding to suspicion of human trafficking. These results demonstrate that, although trained primarily on language, LLMs have internalized biases of human socio-visual perception. Contrary to the promise of enhancing human decisions, these findings expose the recklessness of using language models in selection contexts.

## Introduction

**Figure 1:**
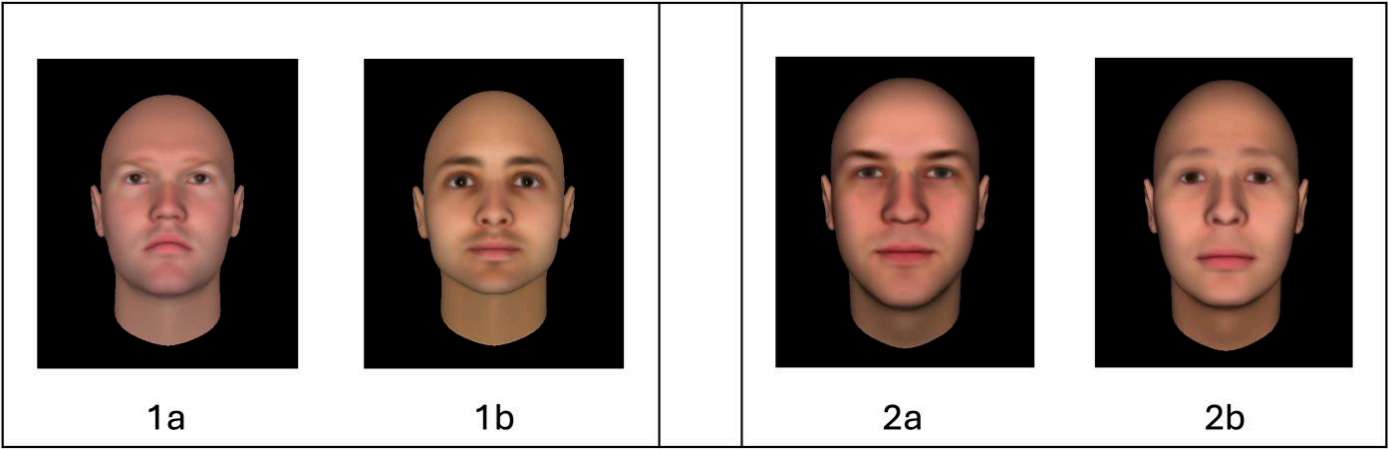
Example faces. Faces 1a and 1b vary on human perceptions of *trustworthiness*, and faces 2a and 2b on human perceptions of *competence*.

Glance at the two faces on the left and decide: which of the two, 1a or 1b, appears to you to be more *trustworthy*? Now, look at the two faces on the right: which of these, 2a or 2b, appears to you to be more *competent*? (Fig. 1)

Humans consistently infer personality or character traits (e.g. *trustworthiness*, *competence*) based solely on facial features (1). Research shows that face 1b, relative to 1a, is typically judged as more *trustworthy* (2). Similarly, face 2a, relative to 2b, is typically judged to be more *competent* (3). Humans draw these inferences quickly, and without resistance: they occur even after only very brief presentations of a face, yet confidence in them only increases with longer stimulus presentation (4, 5). While idiosyncratic individual differences in facial processing also exist (6), specific morphological characteristics reliably elicit character judgments across individuals (2, 7), and these biases have been found consistently, even among the youngest children tested (8). These judgments are also impactful, influencing consequential outcomes like voting behavior (9, 10), sentencing decisions (11, 12), and a variety of workplace outcomes (13–15). And yet, they are widely considered to be *errors*. Research has shown that human facial judgments have little to no basis in reality (1, 16, 17). We asked whether these same errors of character judgment would occur in large language models (LLMs) like GPT-4o.

There are numerous reasons to suppose language models will be immune to face-to-character bias. The multimodal capabilities of these models, including visual perception and analysis, are relatively new, with prior versions trained almost exclusively on language (18–20). It is therefore not presently known whether models like GPT-4o possess the perceptual capabilities necessary to make semantically rich and morally laden assessments of a person's *trustworthiness* and *competence* from only a static 2D image.

Moreover, it is unclear how a model trained primarily on language would even acquire such a bias. While the linguistic record is replete with instances of people inferring character from faces—e.g. “Yond Cassius has a lean and hungry look…such men are dangerous” (21, 1.2.194–195)—it is not intuitively clear that these references contain the level of nuance and specificity that would enable a language-based model to internalize face biases in a manner analogous to humans.

Finally, models like GPT-4o have been carefully trained, using methods like reinforcement learning, to avoid socially undesirable biases (19) and to more generally achieve alignment with human goals and moral sentiment. On judgments that carry an inherent good-bad evaluation (e.g. labeling a person as a liar or unintelligent), LLMs have been trained to strive for neutrality—they tend to avoid making statements that could be harmful (22). If a model behaves in accordance with these alignment objectives, it ought to either show neutrality in judging character from facial configurations, or simply refuse outright to offer judgments of the kind human adults and children render with ease, speed, and confidence. This was a distinct possibility, as there are many instances in which LLMs do refuse to answer. They might, for example, consistently say things like: “Without more information, I cannot gauge which individual is more competent.”

On the other hand, LLMs have increasingly been shown to exhibit unexpected behaviors and abilities for which they were not explicitly trained (18, 23–26). Models trained on vast troves of human knowledge seem to mimic the output of human cognitive processes in deeper and more nuanced ways than previously assumed (26, 27), and face-to-character inference might therefore emerge in them even in the absence of intentional training. Furthermore, early research around GPT-4o's visual reasoning shows surprisingly advanced perceptual capabilities. For example, GPT accurately reads emotions in upright human faces, though, like humans, it is less accurate when faces are inverted (28). Research of this sort suggests that models like GPT may have sufficient perceptual capacities to render humanlike social judgments of faces. Finally, AI models have regularly been shown to reflect and even amplify human biases (29–33), and errors in the accuracy and fairness of AI models are widely acknowledged to pose potent societal dangers (34–37).

Thus, both hypotheses—that LLMs like GPT would show humanlike face-to-character biases or that they would be immune to them—were plausible at the outset. These equal possibilities, and the profound implications either finding would hold for AI decision-making, motivated this research.

One promise of AI has always been its potential to enhance the accuracy and fairness of human decisions. If LLMs are indeed found to be free of face-to-character bias, they will become better candidates for aiding humans on consequential judgments in which the face-to-character inferences of humans impede accuracy and fairness, such as personnel selection and judicial decisions. If the data instead reveal that LLMs like GPT mimic human face-to-character biases, it will be necessary to exercise caution, and implement correctives in their usage as aides to decision-making, given their potential to not only reflect but reinforce and amplify bias.

Across 13 experiments, we tested LLMs’ tendency to make face-based character inferences. GPT-4o completed a series of over 4,500 forced-choice judgments, and other models over 3,400 more, between pairs of faces that varied, in a systematic fashion, on physical features known to influence human judgments of two central character traits: *competence* and *trustworthiness*. In experiments 1 and 2, using stimuli extensively studied with human participants, we assessed whether GPT would mirror human judgments about which of two individuals were more *competent* or *trustworthy* (2, 3). In experiments 3 and 4, to test generalized learning, we explored whether these judgments extended to 12 semantically related traits that have been less studied in humans. In experiment 5, we presented GPT with faces of rhesus macaque monkeys—stimuli for which character judgments do not exist in the LLMs’ training data—and examined whether it drew similar character inferences about them. In two further studies, we examined whether GPT's face-to-character inferences extended to ecologically real and socially meaningful judgments of extreme negative behaviors like serial murder and human trafficking (experiment 6) and consequential positive judgements such as employee selection and worthiness of startup investment (experiment 7). Finally, to assess whether the effects were universal, and if so, whether they grew stronger or weaker with the recent release of advanced chain-of-thought reasoning models, in experiments 8–13, we replicated experiments 1 and 7 with three additional models: GPT-5, Gemini 3 Flash Preview, and Claude Sonnet 4.5.

## Materials and methods

Complete details of the materials and methods, including sample stimuli, exact prompts, and full experimental designs can be found in the Supporting Information.

Across the first seven experiments (including 4,590 trials in total), GPT-4o was presented with pairs of faces in counterbalanced order and asked to select which individual was higher on a given trait (e.g. *competence* or *trustworthiness*). All experiments with GPT-4o were run through OpenAI's API Playground, using the chatgpt-4o-latest model. The computer-generated human faces were drawn from extensive prior research and were designed to vary systematically on human-rated *competence* (3) and *trustworthiness* (2). The images of rhesus macaque monkeys used in experiment 5 were similarly validated in prior human research to appear high or low along a *mean-nice* dimension (38). The same *competence* faces were also used in the six replications with other LLMs (experiments 8–13), which included a total of 3,409 trials.

As described below, all of these experiments except experiment 5 included pairs of faces from arrays that systematically varied the relative degree of physical features associated with human-rated facial *competence/trustworthiness*. Researchers generated these stimuli by collecting human ratings of many faces and using statistical modeling to identify facial shape and texture features associated with trait perceptions. They then morphed base faces along these trait vectors to create versions that were systematically higher or lower in perceived trust/competence, yielding stimuli that were precisely normed on each perceptual dimension (2, 3). As shown in the SI Appendix (Section S11), ChatGPT was unable to identify the human faces or their source, consistent with the conclusion that it did not have direct access to these specific faces within its training data.

**Figure 2:**
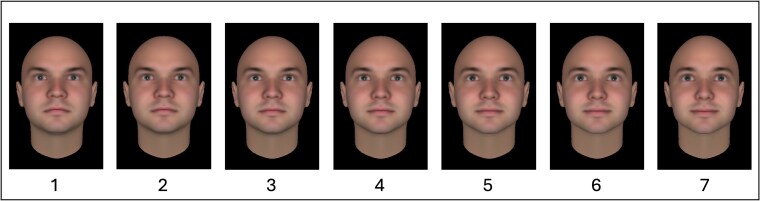
Example array of faces. This array shows features associated with increases in facial *trustworthiness*, such that face 1 tends to be viewed as the least *trustworthy* and face 7 as the most *trustworthy*. This was one of the 10 morphed face identity arrays used in experiments 2, 4, and 6.

Figure 2 illustrates this normed face morphing with a sample array of seven faces, ordered from left to right by features associated with perceived *trustworthiness*, so that face 1 tends to be rated as the least *trustworthy* and face 7 as the most *trustworthy*. Notice that faces that are closer to each other in the array are also more similar in physiognomy—for example, it is more difficult for the human eye to discern the difference between face 3 and face 5 compared to that between face 1 and face 7. If asked to select which person looks more *trustworthy*, over many trials, humans would tend to select face 5 over face 3 but would more effortlessly and consistently select face 7 over face 1. For an LLM to display a truly humanlike bias, it should likewise select more reliably between more distant faces, compared to between the closer ones. In fact, exhibiting this variation in behavior would suggest that the model has acquired a deep conceptualization, however incorrect, of humanlike face-to-character bias.

In experiment 1 (Competence Judgments), GPT-4o made 600 forced-choice decisions (300 each of *competence* and *incompetence*) across 30 pairs of faces—three pairs each drawn from each of 10 morphed face identities like the one above, with one pair differing by 2, one by 4, and one by 6 SD on morphed features associated with facial *competence/incompetence* in human research (3). On each trial, GPT-4o was prompted to select either the more *competent* or the more *incompetent* face. For each of the 30 face pairs, both the order of presentation and the polarity of the trait (*competent* or *incompetent*) were counterbalanced, such that for each pair of faces, GPT made 10 judgments—five with one face presented first, and five with the other presented first—of *competence,* and another 10 (again with five in each presentation order) of *incompetence*. For more direct comparison to human research, a second version of experiment 1 was run for a subset of these faces, using absolute rating scales instead of forced-choice comparisons. This was done to ensure the results of experiment 1 were not an artifact of the forced-choice design. See the SI Appendix for further methodological details on experiment 1 (Section S2) as well as its replication with absolute rating scales (Section S9).

In experiment 2 (Trustworthiness Judgments), GPT-4o made 600 forced-choice selections across 30 pairs of faces that differed by 2, 4, or 6 SD on features associated with perceived facial *trustworthiness/untrustworthiness* in human research (2). On each trial, GPT was prompted to select either the more *trustworthy* or the more *untrustworthy* face. For each of the 30 face pairs, both the order of presentation and the polarity of the trait (*trustworthy* or *untrustworthy*) were counterbalanced, such that GPT-4o made 10 judgments of *trustworthiness* and 10 of *untrustworthiness* for each pair. See the SI Appendix (Section S2) for further methodological details.

In experiment 3 (Competence-related Traits), GPT-4o made a total of 1,080 judgments across six traits conceptually related to *competence*: *Confident*, *Smart*, *Hardworking*, *Lazy*, *Inept*, *and Careless*. GPT judged each of the 30 face pairs—10 each that were 2, 4, or 6 SD apart on features associated with human-rated *competence*—six times each for each of these six traits, with each face pair presented in counterbalanced order. The specific traits used in this study were selected as close semantic associates of the trait *competence*, with that trait itself chosen based on its central role in frameworks like the Stereotype Content Model (39) and on its significance in decades of research on face-to-character judgments (1, 3–5). See the SI Appendix (Section S3) for further methodological details.

In experiment 4 (Trustworthiness-related Traits), GPT-4o made a total of 1,080 judgments across six traits conceptually related to *trustworthiness*: *Warm*, *Helpful*, *Sincere*, *Selfish*, *Hypocritical*, and *Aggressive*. GPT judged each of the 30 face pairs—10 each that were 2, 4, or 6 SD apart on features associated with human-rated *trustworthiness*—six times each for each of these six traits, with each face pair presented in counterbalanced order. As with the *competence* domain, the specific traits used in this study were selected as close semantic associates of the *warmth/trustworthiness* domain. See the SI Appendix (Section S3) for further methodological details.

Experiment 5 (Novel Stimuli) was designed to test whether GPT-4o's face bias would generalize to a type of face unlikely to be evaluated in the model's training data. GPT was presented with 10 images of rhesus macaque monkeys, provided by the lab of Laurie Santos. Five of these faces were previously rated by humans as relatively *mean* and five as relatively *nice*. Each of the five *nice* faces was paired with each of the five *mean* faces, for a total of 25 possible face pairs. GPT-4o made six judgments—with the faces presented in counterbalanced order—for each of these 25 pairs (total *n* = 150 trials), indicating which of the two monkeys was more *trustworthy*. Only one paper to date has reported using these faces for character judgments (38), and these were along the related but distinct dimension of *mean/nice* rather than of *trustworthiness*. Moreover, GPT appeared not to have access to this article in its training data (see SI Appendix, Section S10), and thus could not be expected to have access to its labeled stimuli. It was thus highly unlikely that GPT-4o had been sufficiently trained on this unique combination of stimuli and task, and so, humanlike selections would suggest a deeper representation of this bias, whereby the model applies the morphological features associated with trustworthy human faces to judgments of a different species. See the SI Appendix (Section S4) for further methodological details. These stimuli have been made publicly available at https://osf.io/g69z4/overview.

In experiment 6 (Extreme Negative Behaviors), GPT-4o was presented with the same face pairs used in experiments 2 and 4, which varied in perceived *trustworthiness,* and made a total of 540 judgments across three extremely negative behaviors. Specifically, GPT was asked to indicate which of the individuals was more likely to (i) be a serial killer, (ii) be arrested for human trafficking, and (iii) defraud the public using a Ponzi scheme. GPT judged each of the 30 face pairs—10 each that were 2, 4, and 6 SD apart on features associated with human-rated *trustworthiness*—six times for each of these three behaviors, with each face pair presented in counterbalanced order. The items used in this experiment were specifically selected to be among the most extreme ones to which GPT would respond. A larger pool of items was piloted to ascertain which items GPT would respond to *at all*. However, these data were used only to remove items GPT would not answer, and potentially biased patterns in the model's responses were not examined prior to undertaking the main study. See the SI Appendix (Section S5) for further methodological details.

In experiment 7 (Consequential Real-World Decisions), GPT-4o was presented with the same face pairs used in experiments 1 and 3, which varied in perceived *competence,* and made a total of 540 recommendations around high-impact decisions ostensibly being made by the requesters. Specifically, GPT was asked to indicate: (i) which person we should hire to be the president of a large Midwestern university, (ii) which person's technology startup we should invest in, and (iii) which person we should choose to manage our financial retirement portfolio. GPT judged each of the 30 face pairs—10 each that were 2, 4, and 6 SD apart on features associated with human-rated *competence*—six times for each of these three high-impact decisions, with each face pair presented in counterbalanced order. The specific items used in this experiment were selected as relatively independent examples of high-impact decisions, and were not drawn from a larger pool of piloted items. See the SI Appendix (Section S6) for further methodological details.

Finally, in experiments 8–13, we replicated experiment 1 (competence judgments) and experiment 7 (consequential real-world decisions) with three additional LLMs. These models were: GPT-5, Gemini 3 Flash Preview, and Claude Sonnet 4.5, all examples of the recent generation of advanced chain-of-thought reasoning models. These studies served two purposes. First, they were designed to assess the universality of the reported effects. It was plausible that these effects would prove to be a broader feature of the cognition of advanced LLMs, and equally plausible that they would be a narrower feature, specific to GPT-4o. Second, if face-to-character biases were indeed found across models, these studies were designed to illuminate the relative size of the effects in GPT-4o (the earlier model) compared to these newer and more advanced LLMs. On the one hand, we might predict that their superior reasoning capabilities would enable these models to override facial judgments, since these are known errors, leading to reductions in bias. On the other hand, more advanced models tend to “learn” biases more precisely, and this could lead the more recent models to show even greater bias than GPT-4o. All methodological details of these experiments were identical to those of the prior studies, except that different models were tested, with the data collection completed through their respective APIs. See the SI Appendix (Section S12) for further details.

To analyze the data from each experiment, we examined the proportion of trials in which GPT selected the “expected” face, that is, the one that human participants would typically rate higher, based on prior research using these stimuli. For each analysis, we conducted binomial tests to determine whether the proportion of trials in which GPT selected the expected face differed significantly from chance. Additionally, we used odds ratios (OR) to measure how each selection rate differed from that predicted by chance, and selectively to gauge the effects of other variables (e.g. facial distance) on selection rates. All analyses were conducted in Stata, version 15.1.

### AI disclosure

LLMs (GPT-4o, GPT-5, Claude Sonnet 4.5, Gemini 3 Flash Preview) were the primary subjects of this research. GPT-4o and Claude Opus 4.6 generated scripts for data collection and extraction. GPT-4o, GPT-5.2, and Gemini 3.1 Pro assisted with proofreading, formatting, and analytical recommendations. These LLM tasks were completed under full human supervision. LLMs did not generate any part of this article.

## Results

The results of the seven experiments with GPT-4o are summarized in Table 1. Results of all the experiments, both individually and in aggregate, are described below, with further statistical details in the SI Appendix (Sections S7–9, S12). Except when comparing two different groups or conditions, OR reflect comparisons to chance (50%). In general, OR in the range of 1.5 to 2.5 can be considered small effects. Those in the range of 2.5 to 4 can be considered medium effects. ORs above 4 can be considered large effects.

**Table 1:** GPT-4o's selection of expected faces by experiment and facial distance.

	Number of trials	Expected selections (Combined)	Expected selections (6 SD gap)	Expected selections (4 SD gap)	Expected selections (2 SD gap)
Experiment 1—Competence	600	87.83% *P* < 0.0001	98.00% *P* < 0.0001	95.50% *P* < 0.0001	70.00% *P* < 0.0001
Experiment 2—Trustworthiness	600	72.67% *P* < 0.0001	97.00% *P* < 0.0001	68.00% *P* < 0.0001	53.00% *P* = 0.4368
Experiment 3—Competence-Related Traits	1,080	74.63% *P* < 0.0001	85.56% *P* < 0.0001	81.39% *P* < 0.0001	56.94% *P* = 0.0097
Experiment 4—Trustworthiness-Related Traits	1,080	73.33% *P* < 0.0001	91.39% *P* < 0.0001	71.67% *P* < 0.0001	56.94% *P* = 0.0097
Experiment 5—Trustworthiness (Monkey Faces)	150	66.00% *P* = 0.0001	.	.	.
Experiment 6—Extreme Trust Judgments	540	68.70% *P* < 0.0001	91.11% *P* < 0.0001	62.22% *P* = 0.0013	52.78% *P* = 0.5024
Experiment 7—Consequential Decisions	540	75.19% *P* < 0.0001	89.44% *P* < 0.0001	87.78% *P* < 0.0001	48.33% *P* = 0.7095

This table displays the main results of experiments 1–7, both overall and grouped by the distance between face pairs on features associated with human-rated *competence* or *trustworthiness*. Percentages reflect the proportion of trials in which GPT-4o selected the expected face (i.e. the face that humans would typically rate as higher on a given trait). Statistical significance was calculated using binomial tests comparing the results to chance.

### Experiments 1 and 2

These experiments tested whether, mirroring humans, GPT-4o showed face-to-character biases along two central trait dimensions—*competence and trustworthiness*. Indeed, across trials in experiment 1, GPT selected the expected face (based on human data) as more *competent* 87.83% of the time (OR = 7.219, *P* < 0.0001). In experiment 2, it selected the expected face as more *trustworthy* 72.67% of the time (OR = 2.659, *P* < 0.0001).

Consistent with theory, in both experiments, GPT-4o's selections became less decisive as the distance between the faces diminished, offering machine-based validation of the gradation of the face stimuli developed in the Todorov lab, and evidence of behavior similar to humans. In experiment 1, GPT selected the expected face fully 98.00% of the time (OR = 49.000, *P* < 0.0001) when the faces were 6 SD apart on features associated with human-rated *competence*, and nearly as frequently (95.50%; OR = 21.222, *P* < 0.0001) when they were 4 SD apart. However, when the faces were only 2 SD apart, GPT's selection of the expected face was 70.00%, a rate that, while lower, still significantly exceeded the selection rate expected by chance (OR = 2.333, *P* < 0.0001).

This pattern was even more pronounced when GPT-4o was tasked with selecting more *trustworthy* faces in experiment 2. GPT selected the ostensibly more *trustworthy* face 97% (OR = 32.333, *P* < 0.0001) of the time when the pair was separated by 6 SD on features associated with human *trustworthiness* ratings. However, GPT's selection of the expected face dropped to 68% (OR = 2.125, *P* < 0.0001) when the faces were separated by 4 SD. When the faces were separated by only 2 SD, this fell to 53%, which did not differ significantly from chance (OR = 1.128, *P* = 0.4368).

In experiment 1, we also directly compared GPT-4o's judgments to previously collected human data. This examination revealed that GPT's face-to-character biases were potentially amplified relative to those of humans. Specifically, we examined data from a prior study (3) in which human participants rated the same faces on *competence*, using a 9-point Likert scale. As described in more detail in the SI Appendix (Section S9), we observed GPT's possible bias amplification in two different manners.

First, using established methods based on Thurstone's law of comparative judgment (40, 41), we estimated the proportion of trials in which humans would select the expected face in the same forced-choice task completed by GPT-4o. Across all trials, humans would be expected to select the expected face ∼62.65% of the time, compared to GPT-4o's sharply larger 87.83% (OR = 4.304). This difference was particularly pronounced when the face pairs were more distant in terms of features associated with human-rated *competence*. For face pairs separated by 2 SD, GPT selected the expected face 70.00% of the time, compared to an estimated 60.44% for humans (OR = 1.527). In contrast, when the pairs were separated by 6 SD, GPT's selection rate rose sharply to 98.00%, while estimated human selections rose only slightly to 64.00%, yielding a much greater difference between the two in the odds of selection (OR = 27.563).

Second, we prompted GPT-4o to provide absolute *competence* ratings (*n* = 50 per face) on a 9-point scale—mirroring that used in the human validation study—for the morphs in three of the face identity groups used in experiment 1. This allowed us to more directly compare GPT's *competence* ratings to those of humans, as reported by Oh et al. (3). As detailed in the SI Appendix (Section S9), the linear relationship between face morph level and *competence* ratings across these groups was notably stronger in the data from GPT, relative to human ratings. For example, linear regressions revealed that morph level (i.e. the degree of features associated with human *competence* ratings) explained 81.01% of the variance in GPT's absolute ratings, compared to just 3.59% of the variance in the human ratings.

Because neither of these methods constitutes an exact replication of human studies, these numbers should be viewed as demonstrations rather than precise comparisons between GPT-4o and humans. Nevertheless, differences this sizable—exceeding an order of magnitude in analyses of both forced-choice and Likert-style ratings—are striking, and consistent with the hypothesis that GPT's face-to-character biases around *competence* are amplified relative to those of humans.

### Experiment 3

In experiments 3 and 4, we probed whether GPT's face-to-character biases would generalize to traits that were semantically related to those from experiments 1 and 2, but for which explicitly labeled human data were not available. In experiment 3, when asked about traits semantically related to *competence*, GPT-4o selected the expected face in 74.63% of trials—less frequently than in experiment 1, but still far above chance (OR = 2.942, *P* < 0.0001). As in experiment 1, the model's consistency increased when the face pairs were further apart on features associated with human-rated *competence*. When the faces were 6 SD apart, GPT selected the expected face 85.56% of the time (OR = 5.923, *P* < 0.0001). When the faces were 4 SD apart, this number dropped slightly to 81.39% (OR = 4.373, *P* < 0.0001). When the faces were only 2 SD apart, GPT's selection of the expected face dropped sharply to 56.94%, though this selection rate still significantly exceeded chance (OR = 1.323, *P* = 0.0097). Thus, GPT again exhibited the theoretically predicted humanlike pattern of making the expected selections more reliably when the physiognomic differences between the faces were more pronounced, and yet still inferred trait differences even between pairs of faces that were relatively close together.

### Experiment 4

A similar pattern of generalization was observed for traits semantically related to *trustworthiness*. Here, in contrast to experiment 3, we saw no reduction in GPT's rate of selecting the expected face for the related traits compared to the central trait, *trustworthiness,* itself. Across trials, GPT selected the expected face 73.33% of the time (OR = 2.750, *P* < 0.0001), a rate similar to that observed in experiment 2. While we observed a drop in GPT's rate of selecting the expected face when the faces were closer on human-rated *trustworthiness*, this pattern was actually slightly less pronounced in experiment 4 compared to experiment 2. When the faces were 6 SD apart on features associated with human-rated *trustworthiness*, GPT selected the expected face 91.39% of the time (OR = 10.613, *P* < 0.0001), a slightly lower rate than that observed for judgments of *trustworthiness* itself. This rate fell to 71.67% (OR = 2.529, *P* < 0.0001) when the faces were separated by 4 SD, and to 56.94% when they were separated by 2 SD. Unlike in experiment 2, this last selection rate still differed significantly from chance in experiment 4 (OR = 1.323, *P* = 0.0097). These experimental variations validate that trait judgments correspond to physiognomy in a generalized manner, and also that even small physiognomy differences yield responses from GPT that deviate statistically from chance and reveal face-based bias.

### Experiment 5

In this experiment, we probed the depth of GPT-4o's representation of face-to-character inferences by presenting the model with pairs of faces of rhesus macaque monkeys—stimuli not represented in its training data in the context of character evaluation. If GPT's evaluation of these faces mirrored those of humans, this would provide evidence that the model has learned a physiognomic association to which it has not been directly exposed. These faces did not systematically vary in the distance between different pairs. Instead, they consisted of images of real monkeys that were consistently rated by adults and children as *nicer* or *meaner*. Despite the complete novelty of these stimuli, GPT made selections that aligned with the judgments of humans. Specifically, the LLM selected the faces rated as *nice* by humans as more *trustworthy* in 66.00% of trials, a rate significantly exceeding chance (OR = 1.941, *P* = 0.0001), demonstrating strong (and undesirable) machine-human alignment, while also suggesting that GPT has deeply encoded the morphological characteristics associated with perceived trustworthiness, rather than simply parroting human ratings found in its training data.

### Experiment 6

We tested whether GPT would incorporate perceived facial *trustworthiness* even when we requested extreme judgments of a sort that LLMs are trained to avoid due to concerns about truthfulness and potential harm (42). Specifically, we asked the model to make ascriptions of guilt for serious crimes (murder, human trafficking, and fraud). This was an important test as GPT is known to resist making negative and morally tinged judgments. Across all trials, GPT selected the more *untrustworthy*-looking individual as more likely to be guilty of these offenses 68.70% of the time (OR = 2.195, *P* < 0.0001). The fact that this selection rate was only slightly lower than in experiments 2 and 4 suggests that the LLM was fully willing to apply physiognomy-based inferences even to morally significant and socially consequential judgments of the likely criminality of unknown individuals.

Replicating earlier experiments, this selection rate varied depending on the distance between the faces on features associated with human-rated *trustworthiness*. When the faces were 6 SD apart, GPT selected the expected face 91.11% of the time (OR = 10.250, *P* < 0.0001). This number dropped to 62.22% (OR = 1.647, *P* = 0.0013) when the faces were separated by 4 SD, and to 52.78% when they were separated by only 2 SD, with this last result not statistically distinguishable from chance (OR = 1.118, *P* = 0.5024).

### Experiment 7

This experiment tested the other side of the consequential judgments GPT made in experiment 6, now focusing on positive judgments that reward individuals. Specifically, experiment 7 was designed to test whether GPT-4o's face-to-character bias around *competence* would sway consequential decisions, with positive outcomes for the selected individual, in three high-stakes decisions ostensibly being made by the experimenters: hiring a university president, funding a startup, and selecting a manager for our retirement portfolio.

The results mirrored those of experiments 1 and 3, and were consistent with the results of experiment 6, suggesting that the LLM did not resist incorporating physiognomic biases even into meaningful decisions. Specifically, GPT instructed us to select the more *competent*-looking individual across 75.19% of all trials, a rate significantly above chance (OR = 3.030, *P* < 0.0001). Following the same pattern as prior experiments, this selection rate varied depending upon the distance between the faces on features associated with human-rated *competence*. GPT made the expected selection 89.44% of the time when the faces differed by 6 SD (OR = 8.474, *P* < 0.0001), and nearly as often (87.78%; OR = 7.182, *P* < 0.0001) when they differed by four. However, this rate dropped to chance (48.33%; OR = 0.935, *P* = 0.7095) when the faces differed by only 2 SD.

### Summary of experiments 1–7

Across the seven experiments with GPT-4o, a few key patterns emerged. First and foremost, GPT, like humans, consistently displayed evidence of face-to-character biases. Collapsing across all seven experiments, GPT selected the face that would be expected based on human ratings 74.88% of the time (OR = 2.981, *P* < 0.0001). These results arose reliably across experiments, regardless of whether the LLM was selecting based on the two core traits of *trustworthiness* and *competence* or generalizing to secondary characteristics semantically related to these core traits. Furthermore, as detailed in the SI Appendix (Section S8), all individual effects reported as significant retained this significance after undergoing corrections for multiple comparisons using Holm's sequentially rejective Bonferroni test. We can conclude that the effects obtained in these experiments are highly robust.

In the case of *competence* judgments, the data available from humans suggested that GPT's bias was amplified: our reanalysis of human data estimated a human selection rate of roughly 62.65%, sharply lower than GPT's selection rate of 87.83% in the same task (OR = 4.304). Tellingly, GPT also ascribed even extremely negative attributes like criminality and extremely positive attributes like worthiness of venture capital investment based purely on irrelevant facial features. Finally, the model showed evidence of generalized learning by exhibiting consistent face-to-character bias even when evaluating monkey faces absent from its training data. These results are striking because GPT is an AI model that lacks humanlike awareness and drives and, presumably, has not been trained toward the goal of drawing biased face-to-character inferences. Nevertheless, our data decisively indicate that GPT has developed these physiognomic biases, emergently mirroring the humans that created it.

A variety of secondary findings are also of interest. As detailed in the SI Appendix (Section S7), GPT made the expected selection more consistently for traits that were framed positively (e.g. *competent, hardworking*) compared to those that were framed negatively (e.g. *incompetent, lazy*). Specifically, when collapsing across experiments 1–4, GPT selected the expected face 80.36% of the time (OR = 4.091, *P* < 0.0001) for positively framed attributes, compared to 72.08% for negatively framed attributes (OR = 2.582, *P* < 0.0001). The difference between GPT's hit rates for positively versus negatively framed attributes reached significance in a logistic regression (OR = 1.584, *P* < 0.001). This asymmetry may mirror the human tendency to more easily voice positive social judgments than negative ones (43), again showing machine-human alignment—alignment in these experiments being not a virtue but a shortcoming.

Further, in line with theory and previous data from humans, GPT-4o selected the expected face far more consistently when there was a larger distance between the pairs on features associated with human-perceived *competence/trustworthiness*. For this analysis, we collapsed across experiments 1–4 and 6–7, as these experiments used face pairs that varied in this systematic fashion. On trials where the faces in the pair differed by 6 SD in characteristics associated with *competence/trustworthiness*, GPT selected the expected face fully 91.35% (OR = 10.563), with the results reaching a high threshold for statistical significance (*P* < 0.0001) both in aggregate and within each individual experiment. Results for the intermediate pairs of faces—which differed by 4 SD—were slightly less consistent but still definitive: GPT selected the expected face 77.57% of the time, with the results again reaching significance in aggregate (OR = 3.458, *P* < 0.0001) as well as within each individual experiment (*P* < 0.005). The faces that differed by 2 SD in human-rated *trustworthiness/competence* tended to look visually similar, and it was more difficult for GPT to discern the differences. This provides an indirect test of the validity of GPT's behavior which—as we would expect for humans—is systematically responsive to the physiognomic distance between faces. Even for the faces separated by 2 SD, however, GPT tended on average to select the expected face, with a hit rate above 50% in five of the six experiments that used normed human faces, and reaching statistical significance in half (experiments 1, 3, and 4). Collapsing across the experiments, when judging faces that differed by 2 SD, GPT selected the expected face 56.62% of the time, a result that significantly exceeded chance (OR = 1.305, *P* < 0.0001). As detailed further in the SI Appendix (Section S7), the increase in GPT-4o's rate of selecting the expected face for pairs that were more distant was highly significant: a logistic regression indicated that for each unit increase in face trait distance (from 2 to 4 to 6 SD), the odds of GPT selecting the expected face increased by a factor of 2.802 (OR = 2.802, *P* < 0.001).

### Experiments 8–13 (testing additional models)

Experiments 1–7 focused on GPT-4o, a highly utilized frontier model. While the results with this model were highly consistent, they invite us to consider whether this problem is universal. Will other LLMs show the reported effects, or are they specific to GPT-4o? And, if more recent models *do* show face-to-character biases, how will their magnitude compare to those of GPT-4o? Accordingly, we ran two full studies each with three further LLMs: GPT-5 (experiments 8 and 9), Gemini 3 Flash Preview (experiments 10 and 11), and Claude Sonnet 4.5 (experiments 12 and 13). These studies directly replicated experiments 1 and 7, which had been previously conducted with GPT-4o, except that they instead utilized these three recent and highly advanced chain-of-thought reasoning models. The results of these experiments are summarized in Table 2 (see also SI Appendix, Section S12).

**Table 2:** Experiments with additional LLMs.

	Number of trials	Expected selections (combined)	Expected selections (6 SD gap)	Expected selections (4 SD gap)	Expected selections (2 SD gap)
**Competence judgments**					
GPT-4o: Experiment 1	600	87.83% *P* < 0.0001	98.00% *P* < 0.0001	95.50% *P* < 0.0001	70.00% *P* < 0.0001
GPT-5: Experiment 8	600	94.33% *P* < 0.0001	100.00% *P* < 0.0001	99.00% *P* < 0.0001	84.00% *P* < 0.0001
Gemini 3 Flash Preview: Experiment 10	600	95.67% *P* < 0.0001	100.00% *P* < 0.0001	100.00% *P* < 0.0001	87.00% *P* < 0.0001
Claude Sonnet 4.5: Experiment 12	600	89.00% *P* < 0.0001	92.50% *P* < 0.0001	96.00% *P* < 0.0001	78.50% *P* < 0.0001
**Consequential decisions**					
GPT-4o: Experiment 7	540	75.19% *P* < 0.0001	89.44% *P* < 0.0001	87.78% *P* < 0.0001	48.33% *P* = 0.7095
GPT-5: Experiment 9	540	97.04% *P* < 0.0001	100.00% *P* < 0.0001	100.00% *P* < 0.0001	91.11% *P* < 0.0001
Gemini 3 Flash Preview: Experiment 11	540	87.04% *P* < 0.0001	88.33% *P* < 0.0001	96.11% *P* < 0.0001	76.67% *P* < 0.0001
Claude Sonnet 4.5: Experiment 13	529	89.41% *P* < 0.0001	100.00% *P* < 0.0001	98.86% *P* < 0.0001	69.49% *P* < 0.0001

This table displays the results of experiments 8–13, conducted using three chain-of-thought models, both overall and grouped by the distance between face pairs on features associated with human-rated *competence*. Results from GPT-4o in experiments 1 and 7 are included for comparison. Percentages reflect the proportion of trials in which the relevant models selected the expected face (i.e. the face that humans would typically rate as higher on a given trait). Statistical significance was calculated using binomial tests comparing the results to chance.

One might have expected that the superior reasoning capabilities of GPT-5 would lead it to show less bias than GPT-4o. However, the opposite pattern of results was observed. In experiment 8, GPT-5 actually showed notably *more* physiognomic bias than its predecessor, selecting the expected face as more competent in 94.33% of trials, compared to GPT-4o's 87.83% (OR = 2.306, *P* < 0.001). This pattern was even more pronounced in experiment 9, when GPT-5 advised on consequential decisions. Here, GPT-5 recommended the more competent-looking individual fully 97.04% of the time, sharply more often than GPT-4o's 75.19% (OR = 10.809, *P* < 0.001).

GPT-5's sharply higher bias was particularly clear for the face pairs separated by 2 SD in morphological characteristics. For these closer faces, GPT-5 selected the more competent-looking face 84.00% of the time in experiment 8, compared to GPT-4o's 70.00% in experiment 1 (OR = 2.250, *P* = 0.001). As reported earlier, in experiment 7, when advising on consequential decisions, GPT-4o did not show significant bias on the trials with the 2 SD face pairs, selecting the expected face only 48.33% of the time (*P* = 0.7095). In contrast, in experiment 9, GPT-5 selected the expected face in the 2 SD pairs 91.11% of the time, sharply more often than GPT-4o (OR = 10.957, *P* < 0.001).

The data from Gemini 3 Flash Preview looked more similar to GPT-5 than to GPT-4o. In experiment 10, Gemini selected the expected face as more competent more often (95.67%) than GPT-4o did (87.83%) in experiment 1 (OR = 3.058, *P* < 0.001). Similarly, when advising on consequential decisions in experiment 11, Gemini selected the more competent-looking face 87.04% of the time, significantly more often than GPT-4o (75.19%) did in experiment 7 (OR = 2.216, *P* < 0.001). These patterns were once again particularly pronounced for the faces that were closer together. For these closer face pairs, Gemini selected the expected option as more competent 87.00% of the time in experiment 10, significantly more frequently than GPT-4o (OR = 2.868, *P* < 0.001). For the trials using the same closer faces in experiment 11, Gemini recommended the individual with the more competent-looking face 76.67% of the time when advising on consequential decisions, again significantly more frequently than GPT-4o (OR = 3.512, *P* < 0.001).

On basic judgments of competence, Gemini's selection rate in experiment 10 did not differ from that of GPT-5's rate in experiment 8 (OR = 1.326, *P* = 0.291). However, when advising on consequential decisions in experiment 9, GPT-5 selected the expected face notably more often than Gemini 3 did in experiment 11 (OR = 4.878, *P* < 0.001). This pattern suggests that Gemini 3 holds roughly the same degree of face-to-character bias around competence as does GPT-5. However, Gemini 3 seemingly utilized this bias somewhat less frequently than GPT-5 when advising on consequential decisions, though its degree of bias unquestionably remained problematically large.

In experiment 12, Claude Sonnet 4.5, too, displayed significant bias around basic judgments of competence, but this bias was smaller than that observed for the other chain-of-thought models, and roughly comparable to that observed with GPT-4o. Specifically, Claude selected the more competent-looking face 89.00% of the time, insignificantly more frequently than GPT-4o (OR = 1.121, *P* = 0.528). When making simple judgments of competence, both of the other reasoning models selected the expected face at significantly higher rates than Claude: GPT-5 (OR = 2.058, *P* = 0.001) and Gemini 3 Flash (OR = 2.729, *P* < 0.001). When specifically selecting between the faces that were closest together morphologically, Claude chose the expected face as more competent 78.50% of the time, here, as well, trending toward but not significantly more often than GPT-4o (OR = 1.565, *P* = 0.053). Compared to Claude, GPT-5 descriptively selected the expected face more frequently within these close trials, but the difference was nonsignificant (OR = 1.438, *P* = 0.160). For these same 2 SD trials, Gemini's selection of the expected face was modestly, but significantly, higher than that of Claude (OR = 1.833, *P* = 0.026).

While Claude performed slightly better than the other chain-of-thought reasoning models in experiment 12, this pattern was not seen as consistently in experiment 13, when the model advised on consequential decisions. In this experiment, Claude Sonnet 4.5 selected the expected face 89.41% of the time, more frequently than GPT-4o (OR = 2.788, *P* < 0.001). Here, Claude's bias was roughly equivalent to that of Gemini (OR = 1.258, *P* = 0.229). However, GPT-5's bias in experiment 9 significantly exceeded Claude's bias in experiment 13 (OR = 3.877, *P* < 0.001). A similar pattern was observed when examining only the trials where faces were separated by 2 SD in morphological characteristics. Specifically, when advising on consequential decisions for the 2 SD face pairs, Claude selected the expected face on 69.49% of trials, significantly more frequently than GPT-4o (OR = 2.435, *P* < 0.001), and insignificantly less frequently than Gemini 3 (OR = 0.693, *P* = 0.127). For these closer face pairs, GPT-5 once again made biased judgments notably more frequently than Claude 4.5 (OR = 4.500, *P* < 0.001).

Thus, the following overarching patterns were found in experiments 8–13. On basic judgments of competence, Claude showed roughly the same degree of bias as GPT-4o. In contrast, Gemini 3 and GPT-5 looked similar to each other, and both showed greater bias than either Claude or GPT-4o. However, when advising on consequential decisions, all three chain-of-thought models exhibited significantly more bias than GPT-4o, with GPT-5 showing the most bias of all, higher than its fellow reasoning models, and also sharply higher than its predecessor, GPT-4o.

## Discussion

At the outset, we stated that two opposing outcomes were equally plausible based on previous research. It was possible that LLMs like GPT-4o would be unbiased in their face-to-character assessments or, equally, that they would show humanlike biases on judgments of *trustworthiness* and *competence*. Across seven experiments with GPT-4o and six additional experiments with more recent chain-of-thought models, using 130 different human and primate faces, and obtaining a total of nearly 8,000 trials, we found consistent support for the latter hypothesis.

In experiments 1–2, GPT-4o selected the same faces that humans do as more *competent* and *trustworthy*. In experiment 3, GPT-4o judged faces rated as higher on competence to also be more *Confident*, *Smart*, and *Hardworking*, and less *Lazy*, *Inept*, and *Careless*. Similarly, in experiment 4, the same model indicated that individuals with ostensibly more *trustworthy* faces were also more *Warm*, *Helpful*, and *Sincere*, and less *Selfish*, *Hypocritical*, and *Aggressive*. In experiment 5, GPT-4o applied this face-to-character mapping even to the faces of rhesus macaque monkeys, consistent with the premise that it has generalized patterns of physiognomic inference. In experiment 6, GPT demonstrated a tendency to attribute even extreme negative behaviors—murder, fraud, and human trafficking—to less *trustworthy*-looking human faces. In experiment 7, GPT used perceived facial *competence* as a basis for recommending candidates in high-impact decisions around hiring a university president, funding a tech startup, and selecting an individual to manage an investment portfolio. Finally, in experiments 8–13, GPT-5 was discovered to be even *more* biased than GPT-4o, as were Claude Sonnet 4.5 and Gemini 3 Flash Preview, suggesting that the problem of face-to-character biases is only becoming more pronounced among the most recent generation of chain-of-thought models. The reported patterns were clear and consistent, and survived correction for multiple comparisons (see SI Appendix, Section S8).

The experiments presented in this article focused on judgments of trustworthiness and competence, since these are among the most heavily studied constructs both in social cognition in general, and in face perception research in particular. Future research should extend this work to other central social characteristics, and also to more peripheral traits that have been less studied in human research on person perception, to ascertain whether such biases are a more general feature of LLM social cognition. For example, by also using faces that differ in gender and ethnicity, it should be possible to study whether models rely on group-based stereotypic traits in their judgments, such as rating female faces as more trustworthy compared to equivalent male faces, and judging male faces to be more competent than equivalent female faces. The present data provide initial evidence on the latter count: The faces rated as more competent also appear to be more masculine, according to research with humans (3). Thus, while LLMs tend to avoid direct verbal statements of gender bias (44), physiognomic biases may act as a subtler pathway for biased decisions around gender.

### Bias amplification

The magnitude of the bias obtained with GPT (and the other LLMs) appeared greater than that observed with human participants, at least in the domain of *competence* judgments, where comparable human data were available. This amplification did not appear to purely be an artifact of the two-option forced-choice design, since it replicated using 9-point rating scales—a paradigm closer to that used in most research with human subjects. Furthermore, the later experiments were explicitly designed to test for bias in realistic scenarios, such as simulated employee selection contexts. That said, future research should confirm whether the effects remain both across additional prompt variations and in more ecological conditions and where the model is not explicitly instructed to rely on its instincts when uncertain about the decision. Nevertheless, the substantially larger effect sizes obtained with GPT-4o (and the other LLMs) are suggestive, and consistent with the argument that LLMs are not merely mirroring but also amplifying, this bias.

Why might we see a greater magnitude of bias for LLMs than for humans? This effect, while surprising, is consistent with a broader, growing body of work that shows that AI models tend to exaggerate human characteristics in general, and human biases in particular (26, 27, 29, 32, 33, 45, 46). Thus, the present results may simply reflect this more general tendency of LLMs to amplify biases present in their training data. Understanding the exact nature and source of this amplification—for example, the degree to which it arises from absolute differences in bias versus greater uniformity in responses—is an important direction for future research.

A subtly different—and complementary—interpretation is that GPT has internalized the specific perceptual characteristics that humans use to gauge *competence* in a manner that is more consistent and linear than that of humans themselves. Humans show considerable variability in face-to-character judgments (6), even (and especially) for faces that are more extreme. Indeed, as detailed in the SI Appendix (Section S9), humans did not consistently rate those faces that were highest in characteristics signaling competence as *actually* higher in the trait*—*and often even rated the most extreme faces as less competent than those closer to the center. In contrast, GPT-4o consistently rated these extreme faces as the most competent. However, as discussed in the SI Appendix (Section S12), the extreme linearity of GPT-4o's bias was less pronounced in the experiments with the more recent chain-of-thought reasoning models. These later models, much like humans, sometimes showed less bias for the most distant face pairs, compared to the moderately distant ones. This observation is consistent with the idea that as these models become more advanced, their learning dynamics show a distributional simplicity bias (47). That is to say, they first approximate human competence judgments through broad, linear patterns, before gradually layering on the more complex, nonlinear nuances that characterize actual human judgments.

A third possible interpretation of GPT's apparent bias amplification is related to the dynamics of human rather than LLM judgments. It is possible that the face-to-character biases of human participants are systematically underreported due to the perpetual problem of impression management in psychological research. Both the apparent amplification of GPT's *competence* judgments and the model's willingness to explicitly make decisions around things like criminality—judgments many humans would be reluctant to voice—offer evidence consistent with this possibility. Should this be the case, LLMs’ selections could, paradoxically, offer a clearer view of human biases than data from humans themselves. If this hypothesis proves true across domains in future research, research with LLMs could ultimately prove an invaluable tool for providing less adulterated data about human psychological effects.

### Deep representation of morphological characteristics

The consistency of GPT's judgments across related but distinct attributes (experiments 3 and 4) is telling, as it suggests a generalized bias grounded in facial structure, rather than direct exposure to faces that have been labeled on the relevant traits. On their own, experiments 1 and 2 might have indicated that the LLM was trained on data where these faces were explicitly labeled on *competence* and *trustworthiness*. However, such labeled data do not exist for the related but distinct traits used in experiments 3 and 4. GPT's tendency to generalize face-to-character inferences to these semantically related traits thus points to broader internalization of the conceptual associations underlying these judgments. This observation, like that of bias amplification, must be made somewhat cautiously. The main experiments cannot fully rule out the possibility that GPT has these faces in its training data. If GPT does in fact have access to the labeled faces, it is possible that the results in experiments 3 and 4 instead result from a form of semantic leakage. The faces may be stored with a known trustworthiness or competence label, and these ratings are then transferred to judgments in related domains because these domains are represented as semantically similar to the main traits. However, this interpretation seems unlikely for several reasons.

First, we were not able to find any evidence that GPT was aware of the specific human faces utilized in our studies. In a set of follow-up studies, each human face stimulus used in these studies was individually shared with GPT, with a request that the model identify the face and its provenance (see SI Appendix, Section S11). While the model consistently identified the faces as computer-generated, and even sometimes suggested software that could be used to create them, in no case was the model able to directly identify the stimulus or its source. Indeed, in no instance did the model even raise the possibility that these faces were used to study trust or competence, and similarly, it never mentioned the research of any of the authors involved in the creation of the stimuli. These data suggest that the model did not have direct access to the stimuli or trait labels for them, and thus that it instead momentarily judged the faces based on a representation of human morphological characteristics.

Further evidence of GPT's internalization of face-to-character biases (as opposed to its use of preknown trait labels) comes from the gradation of its responses. GPT made more consistent judgments when the face stimuli were further apart in physiognomy, e.g. for faces that were six compared to 2 SD apart on features associated with *competence* or *trustworthiness*. This consistent pattern suggests that the model was not simply regurgitating direct knowledge of the specific faces. Had the model simply learned these faces on a pixel-by-pixel basis, it should have distinguished the closer faces just as easily as the more distant ones: rote memorization of the faces would lead the model to classify each face uniquely, much like a fingerprint (48, 49). Instead, GPT seems to have acquired continuous representations of these trait dimensions.

Experiment 5 presented further evidence for the generalization of GPT-4o's face-to-character biases. It is reasonable to assume that GPT has assimilated information from its training data about which kinds of human faces are seen as more or less competent and trustworthy, and that this contributes to the effects observed in these studies. It is not likely, however, that the model's training data contain analogous information about facial judgments of rhesus macaque monkeys, and yet GPT made decisions about the trustworthiness of nonhuman primate faces with nearly as much ease and consistency as it did about human faces. This result suggests that GPT, like humans, has integrated information about faces in a quasi-conceptual manner, allowing it to make judgments even of faces for which it cannot be expected to have training data. In short, the model appears to have a representation of human morphological features that are associated with trustworthiness, and applies this representation out-of-sample when asked to gauge the trustworthiness of nonhuman faces.

Taken together, these findings suggest that GPT was not merely retrieving learned labels but has instead developed (or mimics) a conceptual mapping between facial morphology and character, and, in doing so, has internalized the same incorrect associations that underlie human face-to-character biases. In short, the model has potentially developed a conceptual representation of this bias. It should, however, be noted that exactly what such a conceptual representation looks like in an AI model currently eludes scientific understanding.

### Mirroring human characteristics

While GPT-4o's face-based judgments differed from those of humans in some respects—such as the apparent amplification and greater linearity of its competence judgments—several patterns also closely mirrored human behavior. One example, as previously discussed, was the model's greater difficulty in judging between more similar faces. The more similar face pairs are also more difficult to distinguish perceptually, leading humans, like GPT, to less reliably gauge ostensible differences in their competence and trustworthiness, a point also indirectly made by Todorov et al. (50).

Another curiously humanlike pattern was GPT's tendency to draw the predicted judgments less consistently for negatively relative to positively framed attributes. Across experiments 1–4, GPT-4o selected the expected face 80.71% of the time when judging positively valenced traits, compared to 72.14% of the time for negatively valenced traits. This aligns with human behavior, where people, bound by an intrinsic tendency toward politeness, more easily express positive than negative social judgments (43), and similarly tend to discriminate more through helpfulness to one group than harmfulness to another (51). Taken together, these data reflect an eerily humanlike response pattern. It is striking that an AI that has presumably not been explicitly trained to discern differences between human faces should nevertheless internalize subtle differences between them in such a humanlike manner.

### Origins of this bias

Future research should aim to better understand the perceptual and quasicognitive mechanisms underlying GPT's similarity to humans on these tasks, and what elements of training led it to mimic humans in this way—an outcome that was by no means guaranteed. At least two training vectors are possible. We believe it most likely that this bias, though pertaining to images, was learned from natural language. The model's training corpus likely contains innumerable references to more and less trustworthy/competent humans, with these descriptions influenced by facial bias, since the humans who have written them themselves fall prey to it. While such references may seem rather subtle as a source of bias, this interpretation aligns with foundational work on how language corpora transmit humanlike biases (30), with other research on emergence in LLMs (25), and more broadly with the surprising precision with which the transformer networks used to train these models fit the training data (47, 52).

Alternatively, while the stimuli used in our studies do not appear to be in GPT's training data, we cannot rule out the possibility that other labeled faces *are* in these data. It is therefore plausible, albeit perhaps less likely, that the model has learned this bias directly from such labeled stimuli. This would be particularly troubling, as it would suggest that exposure to psychological research on biases and other undesirable human characteristics could give rise to these same characteristics in artificial minds.

### LLMs and generalized egalitarianism

Experiments 6 and 7 yielded some of the most striking results in this series and have crucial implications for ethical AI alignment. The creators of models like GPT-4o have invested substantial efforts into aligning model outputs with human ethical norms, and these efforts have, in some cases, successfully limited the biases these models display in specific domains. For example, while GPT-4o shows strong associations between men and male-dominated professions, it does not also rate male candidates more highly for these professions when advising on hiring decisions (44). This result likely reflects the successful use of domain-specific guardrails to prevent biased behavior around gender. However, our results indicate that these guardrails do not necessarily generalize to lesser-known domains of potential bias. Not only did GPT-4o label faces as more or less *competent* and *trustworthy*; it also extended these judgments to extreme characterizations of the pictured individuals (e.g. as murderers), and even incorporated them into consequential decisions like employee selection (as did the other LLMs). These results point to a critical gap in current alignment approaches: the mitigation of bias appears to be targeted narrowly and at high-profile categories like race and gender and has not extended to subtler and less socially sensitive domains like physiognomy biases. To address this, the developers of language models must adopt broader, domain-agnostic strategies that detect and limit bias wherever it arises, and not just in a few handpicked domains that are likely to elicit public scrutiny.

The results of experiments 6 and 7 also urge caution in using LLMs to automate high-impact decisions. These results suggest that human social biases around physiognomy—and potentially many other lower-profile forms of bias—could be emulated and even amplified by artificial agents built to support or automate such decisions. For this reason, organizations should be cautious about using these models to replace or enhance human-led decisions. If, for example, a defendant or job candidate's face is available to the LLM, face-to-character inferences could impede justice and fairness in its decisions. As LLMs become increasingly integrated into systems with agentic capabilities and decision-making responsibilities, understanding these limitations becomes critical. Without broader alignment strategies, the deployment of these models in high-impact domains risks reinforcing unfair outcomes under the guise of the supposed objectivity and computational rigor of AI systems. That said, the present experiments were conducted under specific experimental conditions. Future research should further establish the degree to which morphological characteristics bias LLM responses in ecological, real-world settings.

While this article focuses primarily on GPT-4o, physiognomic biases appear to be prevalent in other models as well. In replications of experiments 1 and 7, GPT-5, a more current model in the same family, showed this bias to an even greater degree than its predecessor. This likely reflects the tendency for more advanced models to also learn biases more precisely (53) and raises the possibility that as models advance, their biases will only become more entrenched. Furthermore, this bias was discovered also for Gemini 3 Flash Preview and Claude Sonnet 4.5. This pattern is troubling and suggests that physiognomic biases may be a universal feature of advanced multimodal LLMs. Moreover, like GPT-5, these other advanced models displayed, if anything, even greater bias than GPT-4o. While Claude performed slightly better than the other chain-of-thought models, even this supposedly most ethical of models displayed robust and large face-to-character biases. Thus, while model-specific differences in training—such as Claude's unique “Constitutional AI” process (22)—may be somewhat helpful, they are clearly insufficient to achieve generalized egalitarianism.

By establishing physiognomic judgments as a new domain in which LLMs may display bias, the reported findings underscore the need to add audits of these tendencies to established red-teaming efforts. They also point to the likely need for specific guardrails, such as excluding facial images from high-stakes AI decision systems. The extension of other standard mitigation techniques (e.g. constitutional AI, direct instructions, supervised fine-tuning on refusals, counterfactual data augmentation) to physiognomic biases may also be helpful. However, while these interventions may specifically attenuate physiognomic biases, they are unlikely to correct issues in the many other subtle or less publicized domains in which LLMs could display bias. This is a larger problem in the development of ethical AI: The number of potential domains in which models can display harmful behavior is vast, and addressing each one individually is an inefficient—indeed perhaps impossible—task. Our results thus also emphasize the broader need to develop domain-general strategies to train LLMs to recognize and actively embody fairness.

Taken together, the findings in this article provide compelling evidence that GPT-4o and other LLMs make humanlike—and perhaps even more extreme—judgments about individuals based solely on facial structure. The consistency of these patterns across traits and types of faces suggests that models like GPT are not merely parroting these biases, but have internalized them conceptually, and incorporate them even into decisions with obvious consequential outcomes, like judgments of criminality or worthiness of financial investment. Our results highlight the urgent need for alignment strategies that move beyond domain-specific guardrails and toward cultivating a deeper and more domain-generalized sense of fairness in language models. More broadly, our results suggest that LLMs do not simply replicate the sometimes irrational and biased thoughts of humans, but mirror and at times even magnify these patterns in deep and consequential ways.

## Supplementary Material

pgag247_Supplementary_Data

## Data Availability

All data underlying this article, including raw experimental data and transcripts from all LLM conversations, are publicly available on the Open Science Framework (OSF), and can be accessed at: https://osf.io/g69z4/overview (54).
